# Genus *Promalactis* Meyrick (Lepidoptera, Oecophoridae) from China: Descriptions of twelve new species

**DOI:** 10.3897/zookeys.285.4286

**Published:** 2013-04-05

**Authors:** Zhaohui Du, Shuxia Wang

**Affiliations:** 1 College of Life Sciences, Nankai University, Tianjin 300071, P. R. China

**Keywords:** Lepidoptera, Oecophoridae, *Promalactis*, new species, China

## Abstract

Sixteen species of the genus *Promalactis* Meyrick, 1908 from China are described. Among them, twelve species are described as new: *Promalactis bifurciprocessa* **sp. n.**, *Promalactis convexa* **sp. n.**, *Promalactis papillata* **sp. n.**, *Promalactis quadratitabularis* **sp. n.**, *Promalactis quadriloba* **sp. n.**, *Promalactis ramispinea* **sp. n.**, *Promalactis scorpioidea* **sp. n.**, *Promalactis serpenticapitata* **sp. n.**, *Promalactis similiconvexa* **sp. n.**, *Promalactis spinosicornuta* **sp. n.**, *Promalactis strumifera* **sp. n. **and *Promalactis uncinispinea* **sp. n.**; the previously unknown male of *Promalactis dimolybda* Meyrick, 1935 and female of *Promalactis flavescens* Wang, Zheng & Li, 1997 are described for the first time; *Promalactis albipunctata* Park & Park, 1998 and *Promalactis dierli* Lvovsky, 2000 are newly recorded for China. Adults and genitalia are illustrated.

## Introduction

The genus *Promalactis* was established by Meyrick (1908). It currently comprises 179 valid species worldwide, distributed mainly in the Palaearctic and Oriental regions. China has the greatest diversity, with 101 recorded species (Wang et al. 2006, 2009, 2011). This paper presents the results of our recent study of *Promalactis* based on specimens deposited in the Institute of Zoology, Chinese Academy of Sciences, Beijing (IOZ), with some additional specimens from the Insect Collection, College of Life Sciences, Nankai University, Tianjin (NKU). Sixteen species have been identified, including twelve species new for science, and two species new for China.

*Promalactis* is represented by the combination of the following characters: the smooth head with metallic lustre, the lanceolate forewings with various dark or white markings against yellow to deep ochreous brown ground colour; the variously shaped symmetrical or asymmetrical valvae and a narrow to very broad sacculus in the male genitalia; and a developed to ill-defined lamella postvaginalis and an elongate thin ductus bursae in the female genitalia.

Little is known about the biology of this genus. Meyrick (1922) reported that larvae of *Promalactis* fed on rotten wood or bark of Pinaceae and other trees.

## Material and methods

Specimens examined in this study were collected in Anhui, Fujian, Guangdong, Guangxi, Guizhou, Hunan, Jiangxi, Sichuan, Zhejiang Provinces and Xizang Autonomous Region by light traps. Genitalia dissections and slide mounting methods followed Li (2002). Photographs of adults were taken with a Nikon D300 digital camera plus macro lens, and the genitalia were photographed with an Olympus C-7070 digital camera. All the studied specimens, including the types, are deposited in the Insect Collection, the Institute of Zoology, Chinese Academy of Sciences, Beijing, and the Insect Collection of the College of Life Sciences, Nankai University, Tianjin, China.

## Taxonomic accounts

### Promalactis bifurciprocessa sp. n.

urn:lsid:zoobank.org:act:333A38C0-615C-4B6D-90D1-594C4C567610

http://species-id.net/wiki/Promalactis_bifurciprocessa

Figs 1 17

#### Type material.

Holotype ♂ − **China, Anhui Province:** Yungusi, Mt. Huang (30°07'N, 118°11'E), 15.V.1978, coll. Sizheng Wang, genitalia slide No. DZH12198 (IOZ).

#### Diagnosis.

The new species is similar to *Promalactis manoi* Fujisawa, 2002. It can be separated by the left sacculus with distal process bifurcate, the right sacculus with distal process slender and curved ventrad, and the aedeagus with one cornutus in the male genitalia. In *Promalactis manoi*, the distal process of the left sacculus is not bifurcate, the distal process of the right sacculus is broad and curved dorsad, and the aedeagus has two cornuti.

#### Description.

**Figures 1–6.**
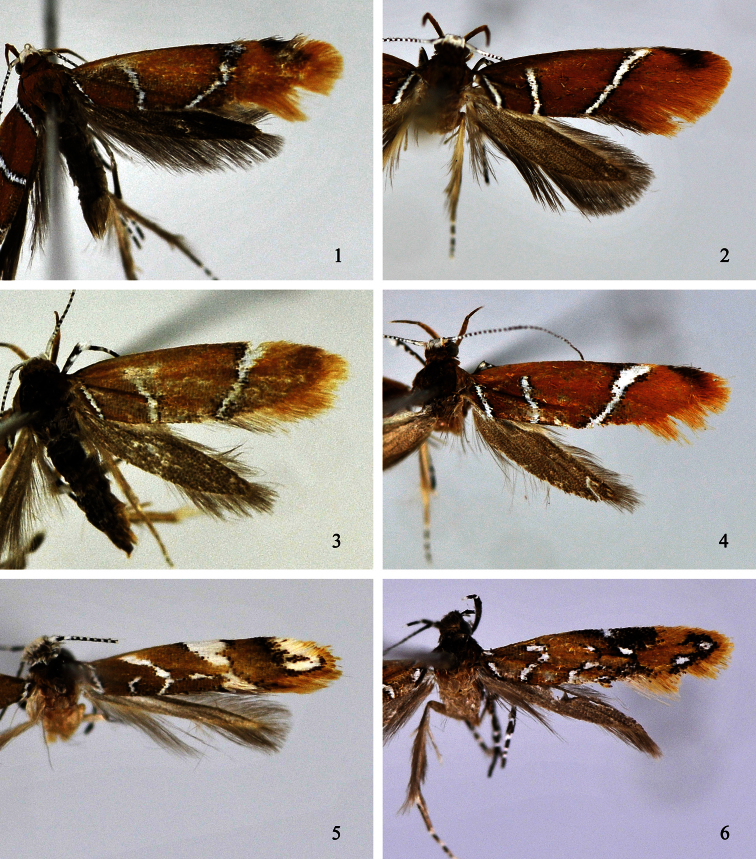
Adults of *Promalactis* species. **1** *Promalactis bifurciprocessa*sp. n., holotype, male **2** *Promalactis convexa* sp. n., holotype, male **3** *Promalactis papillata* sp. n., paratype, female **4** *Promalactis quadratitabularis* sp. n. , holotype, male **5** *Promalactis quadriloba* sp. n., holotype, male **6** *Promalactis ramispinea* sp. n., paratype, female.

Adult (Fig. 1). Wingspan 13.5 mm. Head with vertex shining white, frons dark brown, occiput ochreous brown. Labial palpus with basal and second segments dark orange on outer surface, basal segment light yellow on inner surface, second segment ochreous yellow on inner surface; third segment dark ochreous brown, white at apex, almost same length as second. Antenna with scape white except dark brown on anterior and posterior margins; flagellum white and dark brown on dorsal surface, dark brown on ventral surface. Thorax and tegula ochreous brown. Forewing orange; a narrow white fascia edged with dense black scales from beyond costal 2/3 to before lower angle of cell, then obliquely straight inwards to 3/4 of dorsum, its anterior1/4 widened and densely diffused with black scales; costal margin with an apical blackish brown spot; two white streaks arising from dorsal margin, edged with dense black scales: basal streak from dorsal 1/5 to above base of fold, straight, second streak from dorsal 2/5 to basal 1/3 of upper margin of cell, sinuate, area between two streaks ochreous brown; cilia orange yellow, dark brown along distal part of costal margin. Hindwing and cilia dark grey.

**Male genitalia** (Fig. 17). Uncus heavily sclerotized, nearly trapezoidal, broad at base, slightly narrowed to blunt apex, laterally folded inward and with sparse setae. Gnathos heavily sclerotized, about 3/5 length of uncus, bluntly rounded at apex; lateral arm band shaped, almost same length as gnathos. Tegumen branched from about middle, narrowed anteriorly, blunt apically. Valva asymmetrical; left valva long, slightly narrowed basally, widened distally, rounded apically; costa slightly concave basally, projected distally, rounded apically; sacculus broadened medially, narrowed distally, distal process free, heavily sclerotized, setose, bifurcate distally, forming two spine-like processes: dorsal process curved straight dorsad, apically reaching dorsal 1/4 of valva, ventral process almost straight, apically slightly exceeding end of valva; right valva short, subtriangular, pointed apically, concave inward ventro-distally; costa projected distally; sacculus broad oval, distal process free, very long, curved ventrad, arched inward, far exceeding end of valva, setose distally, acute apically. Saccus short, about 3/5 length of uncus, subtriangular, narrowly rounded at apex. Juxta small, weakly sclerotized, subtriangular. Aedeagus curved, about 1.3 times length of left valva, broad basally, narrowed distally, with a curved, thin apical spine; cornutus spine-like, about 1/4 length of aedeagus, situated near middle of aedeagus.

**Female.** Unknown.

#### Distribution.

China (Anhui).

#### Etymology.

The specific name is derived from Latin *bifurcus* (= bifurcate) and *processus* (= process), referring to the bifurcate distal process of the left sacculus in the male genitalia.

### Promalactis convexa sp. n.

urn:lsid:zoobank.org:act:A53C143D-3AE2-496E-8B69-75E5CE0D542C

http://species-id.net/wiki/Promalactis_convexa

Figs 2 18

#### Type material.

Holotype ♂ − **China, Sichuan Province:** Mt. Qingcheng (30°58'N, 103°31'E), 25.V.1979, genitalia slide No. DZH12027 (IOZ).

#### Diagnosis.

The new species is similar to *Promalactis ermolenkoi* Lvovsky, 1986, but can be separated by the left valva with a beak-like dorso-apical process and the right valva with a hooked dorso-apical process, the left sacculus with a leaf-like distal process and the right sacculus with a spine-like distal process, and the aedeagus with one large cornutus in the male genitalia. In *Promalactis ermolenkoi*, the valva has no dorso-apical process, the left sacculus has a papillary distal process and the right sacculus with an elongate club-shaped distal process, and the aedeagus has two small cornuti. This species is also similar to *Promalactis quadratitabularis* sp. nand *Promalactis similiconvexa*sp. n. The differences between them are stated under each of the latter two species.

#### Description.

Adult (Fig. 2). Wingspan 15.0−16.0 mm. Head with vertex shining white, frons brown, occiput ochreous brown. Labial palpus with basal and second segments orange on outer surface, basal segment light yellow on inner surface, second segment yellow on inner surface; third segment ochreous, slightly shorter than second. Antenna with scape white; flagellum with basal several flagellomeres white, remaining flagellomeres white and black on dorsal surface, black on ventral surface. Thorax and tegula ochreous brown. Forewing ground colour ochreous brown; markings white edged with black scales; a narrow fascia from beyond costal 2/3 extending obliquely inwards to dorsal 3/4, its anterior 2/5 slightly broad; two streaks arising from dorsum: basal streak from dorsal 1/5 extending obliquely to above base of fold, second streak from dorsal 1/3 to above upper margin of cell at basal 1/3; costal margin with a dark brown apical spot; cilia dark orange, dark brown basally at apex, forming a large ill-defined quadrangular spot together with costal spot. Hindwing and cilia dark grey.

**Male genitalia** (Fig. 18). Uncus heavily sclerotized, nearly square, lateral margin arched outward, with sparse setae, posterior margin concave at middle, protruded laterally. Gnathos heavily sclerotized, very short, narrowly banded, distally curved ventrad, with small triangular lateral processes; lateral arm long, heavily sclerotized, about 2/3 length of uncus, band shaped. Tegumen branched from posterior 1/5, slightly narrowed anteriorly. Valva broad, sclerotized, setose distally, asymmetrical; left valva rounded at apex, with a heavily sclerotized, curved, beak-like dorso-apical process, which directs dorsad and bears three teeth distally on outside; sacculus strongly convex dorso-basally, reaching costa posteriorly, then conspicuously narrowed to narrowly rounded apex, with a heavily sclerotized, nearly leaf-like subapical process, which is curved upright, margined with dense teeth, pointed at apex, and reaches middle of dorso-apical process; right valva truncate at apex, with a heavily sclerotized, hooked dorso-apical process, which is upright and pointed at apex; sacculus with basal 3/5 roundly protruding dorso-basally, exceeding costa posteriorly, abruptly narrowed to 3/5, almost same width from 3/5 to 4/5, with a large spine-like process at distal 1/5, distal 1/5 tapered to apex, edged with teeth dorsally. Vinculum nearly triangular, protruding outward latero-medially. Saccus about 3.5 times length of uncus, basal 2/5 broader than distal 3/5, rounded at apex. Juxta roughly oval, weakly sclerotized. Aedeagus curved, about twice length of valva, with a sclerotized, quadrate apical plate; cornutus consisting of some almost coalesced, short, fine spines, forming a large spine, about 1/5 length of aedeagus, situated basally.

**Female.** Unknown.

#### Distribution.

China (Sichuan).

#### Etymology.

The specific name is derived from Latin *convexus* (= convex), referring to the sacculus strongly convex dorso-basally.

### Promalactis papillata sp. n.

urn:lsid:zoobank.org:act:EE5F3D8B-F17C-4C49-A750-C8FF6D610523

http://species-id.net/wiki/Promalactis_papillata

Figs 3 19 31

#### Type material.

Holotype ♂ − **China, Zhejiang Province:** Zhonglieci, Mt. Tianmu (30°19'N, 118°27'E), 400 m, 27.VII.2011, coll. Linlin Yang & Na Chen, genitalia slide No. DZH12147 (NKU); Paratypes − 1 ♂, 3 ♀, same data as holotype except dated 25−27.VII.2011 (NKU). **Anhui Province:** 1 ♀, Julongsi, Mt. Jiuhua, 23.VII.1979, coll. Sizheng Wang (IOZ), genitalia slide Nos. DZH11097 ♀, DZH12137 ♀, DZH12196 ♀, DZH12206 ♂.

#### Diagnosis.

This species is similar to *Promalactis scorpioidea*sp. n.It can be separated by the uncus with two small lateral papillary processes at distal 1/3, and the left sacculus having a strong spine-like process at distal 2/5; the lamella postvaginalis produced to a trapezoidal or quadrangular process on the dorsal surface and to a short quadrangular process on the ventral surface. In *Promalactis scorpioidea* sp. n., the uncus is trilobed distally, the left sacculus has a subrectangular process at distal 1/3; the lamella postvaginalis lacks the process posteriorly. This species is also similar to *Promalactis brevivalvaris* Wang, Li & Zheng, 2000, but the latter can be distinguished by the uncus without papillary process at basal 2/3, with three pointed processes on the posterior margin which are absent in the new species, and the short cornutus about 1/3 the length of the aedeagus, which is 3/5 the length of the aedeagus in the new species.

#### Description.

Adult (Fig. 3). Wingspan 9.0−12.0 mm. Head with vertex shining white, frons and occiput yellowish brown. Labial palpus with basal and second segments ochreous brown on outer surface, light yellow on inner surface; third segment dark ochreous brown, almost same length as second. Antenna with scape white except dark brown on anterior and posterior margins; flagellum white and black on dorsal surface, dark brown on ventral surface. Thorax and tegula ochreous brown. Forewing dark orange yellow, markings white edged with black scales; narrow fascia from costal 2/3 obliquely inwards to end of fold, its anterior 1/2 broad subtriangular; orange yellow from outer margin of fascia to termen; two streaks arising from dorsal margin: basal streak from dorsal 1/5 to base of fold, straight, second streak from dorsal 1/2 to basal 1/3 of upper margin of cell, sinuate; cilia yellow. Hindwing and cilia dark grey.

**Male genitalia** (Fig. 19). Uncus with basal 2/3 broad and parallel sided, with a small, setose, papillary process at basal 2/3 laterally, distal 1/3 narrowed, posterior margin emarginate or narrowly rounded. Gnathos about 3/5 length of uncus, narrow tongue shaped, scobinate, apex narrowly rounded; lateral arm band shaped, slightly shorter than gnathos. Tegumen branched from posterior 1/3, triangularly narrowed anteriorly. Valva with costa slightly concave at base, apex blunt, asymmetrical; left valva almost parallel dorso-ventrally, slight longer than right valva; sacculus broad at base, gradually narrowed to pointed apex, exceeding end of valva, setose medially, strongly dentate and setose along distal 2/5 dorsally, with a heavily sclerotized, strong spine-like process at distal 2/5, which is oblique toward basad; right valva broad basally, slightly narrowed distally; sacculus almost same width except narrowed distally, setose medially, dentate and setose along distal 1/4 dorsally, with a heavily sclerotized, upright, triangular process at distal 1/4, with a small apical spine. Saccus about twice length of uncus, broad at base, slightly narrowed to basal 1/3, distal 2/3 nearly finger-like, rounded at apex. Juxta sclerotized, a large quadrangular plate. Aedeagus curved, about 1.6 times length of left valva, sclerotized distally; cornutus long and curved, spine-like, about 3/5 length of aedeagus.

**Female genitalia** (Fig. 31). Apophysis anterioris about 1/2 length of apophysis posterioris. Lamella postvaginalis large and heavily sclerotized, columniform, sometimes narrowed anteriorly; posteriorly produced to a trapezoidal or quadrangular process on dorsal surface and a short quadrangular process on ventral surface: dorsal process rounded on posterior margin, or concave in V shape at middle and forming two small hill-like lateral processes; ventral process about 2/5 length of dorsal one, slightly concave on posterior margin. Antrum nearly funnelform. Ductus bursae long and coiled, about four times length of corpus bursae, sclerotized except small membranous posterior and anterior sections, dorsally with a sclerotized quadrate plate bearing four curved long spines on right side at posterior 1/6, ventrally with a cluster of short spines at posterior 1/6; ductus seminalis arising from near posterior end of ductus bursae. Corpus bursae rounded, membranous, with dense granules; signum absent.

#### Distribution.

China (Anhui, Zhejiang).

#### Etymology.

The specific name is derived from Latin *papillatus* (= having papillary process), referring to the uncus having a small papillary process at basal 2/3 laterally.

### Promalactis quadratitabularis sp. n.

urn:lsid:zoobank.org:act:86F99BA5-E995-4CE2-A1F7-0B4A781C9720

http://species-id.net/wiki/Promalactis_quadratitabularis

Figs 4 20

#### Type material.

Holotype ♂ − **China, Sichuan Province:** Wanniansi, Mt. Emei (29°32'N, 103°19'E), 14.VI.1979, genitalia slide No. DZH12037 (IOZ). Paratypes − 2 ♂, same data as holotype, genitalia slide Nos. DZH12181, DZH12205 (IOZ).

#### Diagnosis.

This species is very similar to *Promalactis convexa* sp. n., but can be separated by the left valva with an apical spine and a triangular dorso-apical process, the right valva dorsally projected and serrate on distal 1/4, and the sacculus with a triangular distal process on the left and with some distal teeth on the right in the male genitalia. In *Promalactis convexa* sp. n.,the left valva lacks the apical spine and has a beak-like dorso-apical process, the right valva has a hooked dorso-apical process, the sacculus has a leaf-like distal process on the left and a spine-like distal process on the right. *Promalactis pulchra* Wang, Zheng & Li, 1997, *Promalactis similipulchra* Wang, 2006, and *Promalactis zhejiangensis* Wang & Li, 2004 *et al* are externally similar to this new species, but their valva lacks the dorso-apical process on the left, and their narrow sacculus is not strongly convex and does not reach costa posteriorly.

#### Description.

Adult (Fig. 4). Wingspan 14.0−15.0 mm. Head with vertex shining white, frons brown, occiput dark ochreous yellow. Labial palpus with basal and second segments ochreous yellow on outer surface, basal segment light yellow on inner surface, second segment yellow on inner surface; third segment ochreous yellow mixed with dark ochreous brown, almost same length as second. Antenna with scape white except dark brown on anterior and posterior margins; flagellum with basal three flagellomeres white, remaining flagellomeres white and black on dorsal surface, dark brown on ventral surface. Thorax, tegula and forewing dark orange yellow. Forewing with white markings edged with black scales; narrow white fascia from about costal 3/4 obliquely inwards to dorsal 3/4, curved, its anterior 2/5 broadened, with dense diffused dark brown scales on inner margin anteriorly; two streaks arising from dorsum: basal streak from dorsal 1/5 to above base of fold, straight, second streak parallel with basal streak, from dorsal 1/2 to upper margin of cell at basal 1/3, slightly sinuate; costal margin with a apical blackish brown spot; cilia orange yellow, dark ochreous brown basally around apex. Hindwing and cilia ochreous grey.

**Male genitalia** (Fig. 20). Uncus sclerotized, nearly quadrate, shallowly concave at middle on posterior margin, with two small, directing ventrad, triangular processes near posterior margin. Gnathos heavily sclerotized, very short, apically concave at middle, forming two small, triangular lateral processes, curved ventrad; lateral arm about 1.5 times length of gnathos, band shaped. Tegumen branched from posterior 1/4, slightly narrowed anteriorly. Valva broad, sclerotized, setose distally, asymmetrical; left valva having a larger, upright apical spine, with a heavily sclerotized, triangular dorso-apical process directing obliquely basad and serrate dorsally; sacculus strongly convex dorso-basally, slightly exceeding costa posteriorly, conspicuously narrowed to rounded apex, with a heavily sclerotized, serrate, triangular distal process directing obliquely basad, almost same length as and parallel to dorso-apical process of valva; right valva having a smaller, upright apical spine, its distal 1/4 dorsally projected and serrate; sacculus with basal 3/5 roundly protruding, slightly exceeding costa posteriorly, then abruptly narrowed to 3/5, distal 2/5 free, with many heavily sclerotized, ragged dorso-distal teeth, apex narrowly rounded. Vinculum with anterior 1/2 broadened, having a broad transverse band joining lateral sides anteriorly, forming a very short sac antero-ventrally. Saccus elongate, about three times length of uncus, broad at base, gradually narrowed to 2/3, distal 1/3 parallel laterally, rounded at apex. Juxta roughly oval, weakly sclerotized. Aedeagus gently curved, about twice length of valva, slightly dilated basally, with a sclerotized, irregular quadrate plate apically; cornutus consisting of some clustered, almost coalesced fine spines, forming a large, gently curved spine, about 1/5 length of aedeagus, situated basally.

**Female.** Unknown.

#### Distribution.

China (Sichuan).

#### Etymology.

The specific name is derived from Latin *quadratus* (= quadrate) and *tabularis* (= plate shaped), referring to the quadrate apical plate of the aedeagus.

### Promalactis quadriloba sp. n.

urn:lsid:zoobank.org:act:8AE3931A-B02B-4B84-B3DA-1187D5150666

http://species-id.net/wiki/Promalactis_quadriloba

Figs 5 21

#### Type material.

Holotype ♂ − **China, Guizhou Province:** Sanchahe (27°31'N, 106°54'E), Xishui County, 300−500 m, coll. Chunsheng Wu, genitalia slide No. DZH12032 (IOZ).

#### Diagnosis.

This new species is similar to *Promalactis tricuspidata* Wang & Li, 2004, but can be separated by the ventral lobe of the valva having a slender spine-like ventro-basal process, the saccus about the same length as the uncus, the juxta without lateral processes at basal 1/3, the aedeagus without hooked distal process, and the very small cornutus shorter than 1/10 length of the aedeagus in the male genitalia. In *Promalactis tricuspidata*, the ventral lobe of the valva lacks ventral process, the saccus is about four times the length of the uncus, the juxta has lateral processes at basal 1/3, the aedeagus has a hooked distal process, and the long cornutus is about 1/4 length of the aedeagus in the male genitalia.

#### Description.

Adult (Fig. 5). Wingspan 9.0−9.5 mm. Head milk white, occiput white tinged with ochreous brown. Labial palpus with basal and second segments grey on inner surface, brown on outer surface, second segment black at apex; third segment yellow mixed with black except white at base and apex, slightly shorter than second. Antenna with scape white, pecten yellowish brown; flagellum white and black on dorsal surface, black on ventral surface. Thorax and tegula dark yellowish brown. Forewing ground colour yellowish brown; markings white edged with black scales; costal margin black along basal 1/4, with a slender fascia from base extending to dorsal margin, with a broad streak extending from subcostal 1/6 obliquely to middle of fold, with a large patch at costal 1/2 extending downward to near end of cell, contracted latero-medially, bearing dense black scales antero-laterally; dorsal margin with a V-shaped pattern extending from before 1/3 to before 2/5 of fold, with a L-shaped pattern from 1/2 straight outward to middle of fold, then curved outward to before lower angle of cell; apex with a large ovate spot, mixed with black scales, edged with dense black scales except on anterior margin; an irregular spot before tornus, extending upward to lower angle of cell; cilia ochreous yellow. Hindwing and cilia grey.

**Male genitalia** (Fig. 21). Uncus with basal 3/5 slightly wide, sclerotized laterally, distal 2/5 sclerotized, compressed laterally, apex pointed and curved ventrad; laterally with a long, strong setae at basal 2/5. Gnathos rectangular, straight at apex, about 3/5 length of uncus; lateral arm broad, subtriangular, about same length as gnathos. Tegumen broad posteriorly, branched from near posterior margin, rounded anteriorly. Valva narrowed basally, broadened distally; apex with three slender lobes: dorsal lobe heavily sclerotized, curved, with an apical tuft of setae directing ventrad; median lobe with basal 3/4 slender, distal 1/4 expanded and setose, slightly exceeding end of dorsal lobe, directing obliquely dorsad, close to dorsal lobe at base; ventral lobe with basal 3/5 broad triangular, distal 2/5 slender digitate, bearing a spine-like process ventro-basally, with tufted hairs apically, slightly shorter than median lobe. Costa sclerotized, broad at base, gradually narrowed distally. Sacculus indistinct. Saccus broad at base, gradually narrowed to rounded apex, about same length as uncus. Juxta very narrow at base, gradually broadened to about middle, sclerotized laterally; distal half bilobed, heavily sclerotized, arched outwards, obliquely truncate at apex, reaching anterior 2/5 of tegumen. Aedeagus slightly arched, about 1.3 times length of valva, triangular distally; cornutus very small, shorter than 1/10 length of aedeagus, spine-like, situated at about middle of aedeagus.

**Female.** Unknown.

#### Distribution.

China (Guizhou).

#### Etymology.

The specific name is derived from the Latin prefix *quadri-* (= four), and the suffix -*lobus* (= lobe), referring to the three apical lobes and the ventral process of the valva.

### Promalactis ramispinea sp. n.

urn:lsid:zoobank.org:act:9DB7D7E5-27D1-4912-83E5-52D0362CA290

http://species-id.net/wiki/Promalactis_ramispinea

Figs 6 22 32

#### Type material.

Holotype ♂ − **China,** **Jiangxi Province:** Mt. Lu (26°30'N, 115°58'E), 382.8 m, 1.VIII.1975, coll. Youqiao Liu, genitalia slide No. DZH11025 (IOZ). Paratypes − 1 ♀, same data as holotype except dated 9.VII.1975; 4 ♂, 5 ♀, same data as holotype except dated 28.VII−1.VIII.1975. **Jiangxi Province:** 2 ♂, 1 ♀, Xingguo County (26°19'N, 115°20'E), 4, 19, 21.VII.1976; 1 ♀, Mt. Wuyi, 670 m, 2.VIII.1980, genitalia slide Nos. DZH12012 ♂, DZH12013 ♀ (IOZ). **Hunan Province:** 3 ♀♀, Cangxi Town, Xinhua County (27°44'N, 111°18'E), 8−9.VIII.2004, coll. Yunli Xiao. **Fujian Province:** 1 ♀, Guadun (27°44'N, 117°38'E), Mt. Wuyi, 1100 m, 29.VII.2008, coll. Weichun Li, Yongling Sun & Haiyan Bai. **Guangdong Province:** 1 ♀, NanLing (23°20'N, 115°23'E), Shaoguan City, 7−14.VII.2007, coll. Min Wang *et al*., genitalia slide Nos. DZH12043 ♀, DZH12044 ♀, DZH12045 ♀ (NKU).

#### Diagnosis.

This species is similar to *Promalactis trapezia* Wang, 2006, but can be separated by the forewing with a white spot on termen; the tongue-shaped gnathos, the valva with a thick, curved digitate dorso-apical process, and the cornutus distally bearing four to five strong spines in the male genitalia. In *Promalactis trapezia*, the forewing has no white spot on termen; the gnathos is somewhat trapezoidal, the valva has some strong dorso-apical spines and the cornutus is a single spine.

#### Description.

Adult (Fig. 6). Wingspan 10.0−12.0 mm. Head shining greyish brown. Labial palpus with basal and second segments yellowish grey on inner surface, dark brown on outer surface; third segment with basal 1/4 and distal 1/4 white, middle 1/2 black, about 3/5 length of second. Antenna with scape black mixed with white on dorsal surface, yellow on ventral surface, pecten dark brown; flagellum white and black on dorsal surface, yellow on ventral surface. Thorax and tegula ochreous brown. Forewing ground colour orange yellow; costal margin with an inverted triangular black blotch at basal 3/5, posteriorly crossing half wing, with a small white spot at middle within black blotch; cell with a very short, longitudinal white streak at 1/3 on upper margin, with a small white spot at 3/4 and near outer margin; fold with a short white streak at base, a rectangular spot above 1/3 sometimes connected with the white streak at 1/3 of cell, and a L-shaped white streak above 2/3; dorsal margin with three white streaks arising from basal 1/6, 1/3 and 1/2 reaching obliquely to fold respectively, median streak sometimes joined with the spot above 1/3 of fold, third streak sometimes connected with L-shaped streak, with a sinuate weak white line from distal 1/3 to end of fold; tornus with a diffused triangular black spot, extending upward to lower angle of cell; apex and termen with a white spot respectively, surrounded with dense black scales; cilia yellow, tinged with white scales at tornus. Hindwing and cilia dark grey.

**Male genitalia** (Fig. 22). Uncus elongate triangular, broad at base, narrowed to narrowly rounded apex. Gnathos tongue shaped, about same length as uncus, distal 1/3 scobinate; apex rounded, with a small papillary process; lateral arm short, band shaped. Tegumen branched from posterior 2/5, triangularly narrowed anteriorly. Valva almost parallel dorso-ventrally; apex obliquely truncate, dorso-apical process thick digitate, curved ventrad, forming a right angle with apex, with sparse setae distally, blunt at apex; costa straight except slightly projected subapically. Sacculus about 2/5 width of valva, slightly narrowed to a short free distal process, distal 2/3 setose. Saccus broad, triangular, about same length as uncus. Juxta weakly sclerotized, extremely broad, nearly oval, reaching anterior 1/5 of tegumen, with a small saccate basal process, with digitate lateral processes at distal 2/5. Aedeagus strong, almost straight, nearly as long as valva, basal 3/5 membranous, distal 2/5 heavily sclerotized; cornutus strong and curved, about 1/2 length of aedeagus, slightly dilated near base, slender medially, distal 1/4 with four to five strong spines.

**Female genitalia** (Fig. 32). Apophysis anterioris about 1/2 length of apophysis posterioris, apophyses anterioris and posterioris expanded distally. Eighth sternum very short, rounded posteriorly. Seventh sternum slightly concave medially on posterior margin, posterior 1/5 sclerotized, laterally produced to a sclerotized, curved, gradually narrowed band. Antrum concave at middle on posterior margin, protruded in a short triangle postero-laterally, heavily sclerotized laterally; left side with anterior half concave inward, produced to a broad folded band stretching to ductus bursae. Ductus bursae long and curved, about 2.5 times length of corpus bursae, with a sclerotized shield-like plate at middle; posterior 2/5 sclerotized, with fourteen small spines posteriorly; anterior 3/5 membranous; ductus seminalis arising from posterior 1/4 of ductus bursae. Corpus bursae membranous, nearly rounded; two signa small, irregular oval.

#### Distribution.

China (Fujian, Guangdong, Hunan, Jiangxi).

#### Etymology.

The specific name is derived from the Latin prefix *rami-* (= ramus), and Latin *spineus* (= spine-like), referring to the strong spines in the distal 1/4 of the cornutus.

**Figures 7–12.**
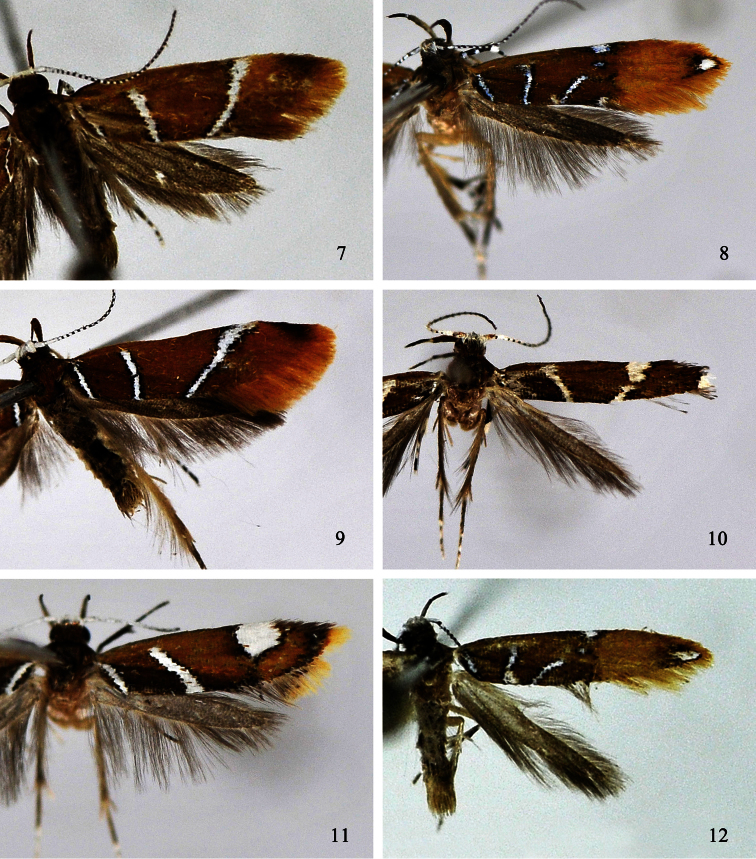
Adults of *Promalactis* species. **7** *Promalactis scorpioidea* sp. n., holotype, male **8** *Promalactis serpenticapitata* sp. n., paratype, female **9** *Promalactis similiconvexa* sp. n., holotype, male **10** *Promalactis spinosicornuta* sp. n., holotype, male **11** *Promalactis strumifera* sp. n., holotype, male **12** *Promalactis uncinispinea* sp. n., holotype, male.

### Promalactis scorpioidea sp. n.

urn:lsid:zoobank.org:act:6261C8FD-0583-4A6B-9776-B437CB0C59A1

http://species-id.net/wiki/Promalactis_scorpioidea

Figs 7 23 33

#### Type material.

Holotype ♂ − **China, Jiangxi Province:** Mt. Lu (26°30'N, 115°58'E), 335 m, 1.VII.1975, coll. Youqiao Liu, genitalia slide No. DZH12188 (IOZ). Paratypes − 1 ♂, same data as holotype; 1 ♂, 1 ♀, same data as holotype except dated 26.VI.1975; 1 ♀, same data as holotype except dated 30.VI.1975; 1 ♂, 1 ♀, same data as holotype except dated 28.VII.1975, genitalia slide Nos. DZH12030 ♂, DZH12187 ♂, DZH12189 ♀, DZH12190 ♀, DZH12191 ♀, DZH12192 ♂ (IOZ).

#### Diagnosis.

This species is similar to *Promalactis tridentata* Wang & Li, 2004, but can be separated by the sacculus distally curved like a scorpion tail and the cornutus about 1/2 the length of the aedeagus in the male genitalia; and the columniform lamella postvaginalis in the female genitalia. In *Promalactis tridentata*, the sacculus is nearly straight distally and the cornutus is about 1/3 the length of the aedeagus in the male genitalia; and the lamella postvaginalis is bell shaped in the female genitalia. This species is also similar to *Promalactis papillata* sp. n. The differences between them are stated under the latter species.

#### Description.

Adult (Fig. 7). Wingspan 11.5−13.5 mm. Head with vertex shining white, frons yellowish brown, occiput ochreous brown. Labial palpus with basal and second segments ochreous brown on outer surface, basal segment yellowish white on inner surface, second segment yellow on inner surface; third segment dark ochreous brown except white at apex, almost same length as second. Antenna with scape white except dark brown on anterior margin; flagellum white and black on dorsal surface, dark brown on ventral surface. Thorax and tegula ochreous brown. Forewing ochreous brown to ferrugineous, costal margin black along basal 1/4; markings white edged with black scales; a narrow white fascia from costal 2/3 obliquely inwards to end of fold, broadened anteriorly, inner margin with diffused dense black scales anteriorly; two white streaks arising from dorsal margin: basal streak from dorsal 1/5 to above base of fold, second streak parallel with basal streak, from dorsal 2/5 to above basal 1/3 of upper margin of cell, widened; cilia dark orange, dark ochreous brown along distal part of costal margin. Hindwing and cilia dark grey.

**Male genitalia** (Fig. 23). Uncus short, narrow at base, broadened slowly, trilobed distally: lateral lobes digitate, setose, middle lobe thicker and longer than lateral lobes. Gnathos almost same length as uncus, very narrow, scobinate, apex rounded; lateral arm band shaped, broad at base, about 4/5 length of gnathos. Tegumen narrowed posteriorly, branched from posterior 1/3, blunt anteriorly. Valva irregularly rectangular, setose distally, apex blunt; costa slightly concave at base. Sacculus broadened near base, narrowed distally, distal 1/5 free, curved dorsad in a right angle, like tail of a scorpion; slightly asymmetrical: left sacculus with distal 1/3 heavily sclerotized, dentate on dorsal margin, with a large heavily sclerotized subrectangular process at distal 1/3, which directs basad and bears some teeth on dorsal margin; right sacculus with distal 3/10 heavily sclerotized, dentate on dorsal margin, with a heavily sclerotized subtriangular process at distal 3/10, which directs basad and bears large teeth dorsally. Saccus about twice length of uncus, broad at base, slightly narrowed to basal 1/3, distal 2/3 nearly finger-like, rounded at apex. Juxta weakly sclerotized, roughly oval. Aedeagus slightly curved, about twice length of valva, sclerotized distally; cornutus long and curved, spine-like, about 1/2 length of aedeagus.

**Female genitalia** (Fig. 33). Apophysis anterioris about 1/2 length of apophysis posterioris. Lamella postvaginalis large and heavily sclerotized, columniform, narrowed anteriorly, broadened posteriorly, posterior margin sinuate, anterior margin heavily concave medially and expanded laterally on dorsal surface. Ostium bursae large. Antrum very short. Ductus bursae long and coiled, about four times length of corpus bursae, sclerotized except small membranous posterior and anterior sections, dorsally with a sclerotized quadrate plate bearing five curved long spines on right side at posterior 1/6, ventrally with a cluster of short spines at posterior 1/6; ductus seminalis arising from near posterior end of ductus bursae. Corpus bursae rounded, membranous, with dense granules; signum absent.

#### Distribution.

China (Jiangxi).

#### Etymology.

The specific name is derived from Latin *scorpioideus*(= like tail of a scorpion), referring to the sacculus curved distally like the tail of a scorpion.

### Promalactis serpenticapitata sp. n.

urn:lsid:zoobank.org:act:DCB54EEC-278B-459D-9C12-06978B25B493

http://species-id.net/wiki/Promalactis_serpenticapitata

Figs 8 24 34

#### Type material.

Holotype ♂ − **China,** **Fujian Province**: Sangang (27°45'N, 117°40'E), Mt. Wuyi, 740 m, 25.VII.2008, coll. Weichun Li, Yongling Sun & Haiyan Bai, genitalia slide No. DZH12055 (NKU). Paratypes − 6 ♂, 18 ♀, Guadun (27°44'N, 117°38'E), Mt. Wuyi, 1100 m, 28.VII−2.VIII.2008, coll. Weichun Li, Yongling Sun & Haiyan Bai. **Zhejiang Province:** 2 ♀, Qingliang Peak (30°07'N, 118°51'E), Linan City, 900 m, 8, 12.VIII.2005, coll. Yunli Xiao; 1 ♀, Sanmuping, Mt. Tianmu (30°26'N, 119°34'E), 1000 m, 29.VII.2011, coll. Linlin Yang & Na Chen, genitalia slide Nos. W04148 ♀, DZH12046 ♂, DZH12047 ♀, DZH12048 ♀ (NKU). **Jiangxi Province:** 1 ♀, Xiaoxidong II (26°28'N, 114°11'E), 5.VII.1978, genitalia slide No. DZH12038 ♀ (IOZ).

#### Diagnosis.

This new species is similar to *Promalactis maculosa* (Wang & Li, 2001), but can be separated by the forewing without white streak on the cell from basal 1/3 to middle; the distal process of the sacculus nearly L shaped and far exceeding the tip of the costa, the nearly rod-like juxta without lateral lobes and the aedeagus with a heavily sclerotized distal process and one cornutus in the male genitalia. In *Promalactis maculosa*, the forewing has a white streak on the cell from basal 1/3 to middle, the distal process of the sacculus is digitate and not exceeding the tip of the dorso-apical process, the juxta has strong lateral lobes and the aedeagus has no distal process and has two cornuti in the male genitalia. This species is also similar to *Promalactis uncinispinea*sp. n. The differences between them are stated under the latter species.

#### Description.

Adult (Fig. 8). Wingspan 10.5−13.0 mm. Head dark brown, vertex white or lateral sides white only. Labial palpus with basal and second segments dark brown on outer surface, basal segment pale white on inner surface, second segment yellowish grey on inner surface; third segment black except white at base and apex, about same length as second. Antenna with scape white except black on anterior and posterior margins; flagellum black, with white annuli on dorsal surface. Thorax and tegula dark ochreous brown, tinged with dark brown scales. Forewing with basal 3/5 ochreous brown, distal 2/5 ochreous yellow; markings silvery white or white, edged with dense black scales; costal margin with a semicircular or quadrate silvery white spot at middle; cell with a small silvery white dot on upper margin under costal spot; three silvery white streaks arising from dorsal margin: basal streak to base of fold, second streak from dorsal 1/3 straight to basal 1/3 of cell, third streak from dorsal 3/5 obliquely to distal 1/4 of cell on lower margin; fold with a white dot at end; apex with an elliptic white spot, edged with dense black and ochreous brown scales; cilia ochreous yellow, grey along distal part of dorsal margin. Hindwing and cilia dark grey.

**Male genitalia** (Fig. 24). Uncus subtriangular, broad at base, gradually narrowed to rounded apex, with a subapical tooth. Gnathos about 3/5 length of uncus, broad at base, gradually narrowed to 2/3, distal 1/3 broadened and rounded, ventrally with a small, snake head-shaped subapical process; lateral arm band shaped, about 2/3 length of gnathos. Tegumen narrow posteriorly, convex laterally at posterior 1/3, branched from posterior 2/3, anterior 1/3 nearly parallel sided, rounded apically. Valva sclerotized except an ovate membranous area medially before apex; basal 2/3 almost parallel dorso-ventrally, distally produced to a setose papillary process; costa concave basally and distally, slightly projected at middle. Sacculus broad at base, slightly narrowed distally, concave between 1/2−2/3 dorsally, distal 1/3 produced to a free, setose, L-shaped distal process, directing dorsad, apically serrate and far exceeding tip of costa. Vinculum widened anteriorly, with a slender transverse band joining left and right sides, forming a fan-shaped area between this band and posterior margin of saccus. Saccus short and broad, about 3/4 length of uncus, subtriangular, pointed at apex. Juxta long, nearly rod-like, slightly curved, with a short digitate basal process, distal 7/10 with a bundle of setae on dorsal surface, with longer setae on distal 2/3, apically with dense spinules or teeth, reaching near middle of uncus. Aedeagus straight and strong, about 4/5 length of valva; with two pieces of dense microtrichia and a heavily sclerotized plate distally, basal half of the plate thick and somewhat conical, distal half spine-like and curved; cornutus spine-like, situated at middle, about 1/3 length of aedeagus, with three short spines and one triangular plate basally.

**Female genitalia** (Fig. 34). Apophysis anterioris stronger than and about 1/2 length of apophysis posterioris. Eighth abdominal segment very short, sternum heavily sclerotized postero-medially, rounded on posterior margin. Seventh abdominal segment sclerotized. Antrum large, inverted trapezoidal, sclerotized except an oval membranous area anteriorly on left side, slightly convex at middle on posterior margin ventrally, lateral margin sinuate. Ductus bursae membranous, slightly longer than corpus bursae, with some short spines posteriorly; ductus seminalis arising from near antrum. Corpus bursae large, nearly rounded, membranous; signum absent.

#### Distribution.

China (Fujian, Jiangxi, Zhejiang).

#### Etymology.

This specific name is derived from the Latin prefix *serpent-* (= snake-like), and the adjective *capitatus* (= having a head), referring to the small, snake head-shaped subapical process on the ventral surface of the gnathos.

### Promalactis similiconvexa sp. n.

urn:lsid:zoobank.org:act:B883343D-DAE4-4B08-AB08-48058EEBFF5D

http://species-id.net/wiki/Promalactis_similiconvexa

Figs 9 25

#### Type material.

Holotype ♂ − **China, Sichuan Province:** Mt. Qingcheng (30°58'N, 103°31'E), 24.V.1979, genitalia slide No. DZH12178 (IOZ).

**Diagnosis.** This species is extremely similar to *Promalactis convexa* sp. n. It can be separated by the left valva with a small hill-like apical process, the left sacculus with distal process reaching basal 1/4 of dorso-apical process of the valva; the right valva with a large quadrate dorso-apical process dentate apically, and the right sacculus with a small subtriangular distal process in the male genitalia. In *Promalactis convexa* sp. n., the left valva is rounded at apex and lacks the apical process, the distal process of the left sacculus reaches the middle of the dorso-apical process of the valva; the right valva has a hooked dorso-apical process and the right sacculus has a spine-like distal process. This species is also externally similar to *Promalactis baotianmanensis* Wang, Li & Zheng, 2000, *Promalactis guangxiensis* Wang, 2006 and *Promalactis parki* Lvovsky, 1986 *et al*., but can be easily separated by the valva having a dorso-apical process, which is absent in each of the latter three species.

#### Description.

Adult (Fig. 9). Wingspan 15.5 mm. Head with vertex shining white, frons brown, occiput dark ochreous brown. Labial palpus with basal and second segments ochreous brown on outer surface, basal segment light yellow on inner surface, second segment ochreous yellow on inner surface; third segment dark ochreous brown, white at apex, shorter than second. Antenna with scape white except dark brown on anterior and posterior margin; flagellum with basal three flagellomeres white, remaining flagellomeres white and black on dorsal surface, dark brown on ventral surface. Thorax and tegula ochreous brown. Forewing ochreous brown; markings white edged with black scales; a narrow white fascia from costal 3/4 obliquely inwards to dorsal 3/4, anterior 2/5 broadened, inner margin with diffused dense black scales anteriorly; two white streaks arising from dorsal margin: basal streak from dorsal 1/5 to above base of fold, second streak from dorsal 2/5 to basal 1/3 of upper margin of cell, slightly arched, area dark ochreous brown between these two streaks; costal margin black along basal 1/4, with a blackish brown apical spot; cilia ochreous brown, dark brown along distal part of costal margin. Hindwing and cilia dark grey.

**Male genitalia** (Fig. 25). Uncus heavily sclerotized, nearly square, lateral margin arched outward, with sparse setae, posterior margin concave at middle, protruded laterally. Gnathos heavily sclerotized, about 1/2 length of uncus, apex curved ventrad, concave at middle, forming two small triangular lateral processes; lateral arm subtriangular, almost same length as gnathos. Tegumen branched from posterior 1/5, triangularly narrowed anteriorly. Valva sclerotized, setose distally, asymmetrical; left valva with apex dentate, with a small hill-like apical process, with a heavily sclerotized, broad beak-like dorso-apical process, which directs obliquely dorsad and is serrate dorso-medially; sacculus strongly protruding basally, reaching costa posteriorly, with a heavily sclerotized, nearly thorn-like subapical process directing dorsad and slightly curved inward, serrate marginally, apically pointed and reaching basal 1/4 of dorso-apical process of valva; right valva quadrate and slightly curved inward distally, apex dentate, with two larger teeth; sacculus with basal 2/3 roundly protruding dorsad, exceeding costa posteriorly, abruptly narrowed to 2/3, almost same width from 2/3 to 5/6, distal 1/6 gradually narrowed to narrowly rounded apex, with a small, heavily sclerotized, subtriangular process at distal 1/6, which is dentate on inner margin. Vinculum nearly triangular, widened latero-medially. Saccus about 2.7 times length of uncus, basal 2/5 broader than distal 3/5, rounded at apex. Juxta roughly oval, weakly sclerotized. Aedeagus slightly curved, about twice length of valva, apex with a sclerotized, quadrate plate; cornutus consisting of some almost coalesced, short, fine spines, forming a large curved spine, shorter than 1/5 length of aedeagus, situated basally.

**Female.** Unknown.

#### Distribution.

China (Sichuan).

#### Etymology.

The specific name is derived from the Latin prefix *simili*- (= similar), and the species name *convexa*, referring to the similarity of the two species.

### Promalactis spinosicornuta sp. n.

urn:lsid:zoobank.org:act:51545D2D-88D5-4CA1-B8E9-148D445AD89C

http://species-id.net/wiki/Promalactis_spinosicornuta

Figs 10 26

#### Type material.

Holotype ♂ − **China, Xizang Autonomous Region:** Motuo County (29°13'N, 95°18'E), 1080 m, 21.VIII.2006, coll. Fuqiang Chen, genitalia slide No. DZH12011(IOZ). Paratype − 1 ♂, same data as holotype, genitalia slide No. DZH12009 (IOZ).

#### Diagnosis.

This new species is similar to *Promalactis ruiliensis* Wang, 2006, but can be separated by the forewing without white dot on termen; the bifurcate part of the uncus curved ventrad, the costa without strong distal spines, and the aedeagus with numerous short spinose cornuti. In *Promalactis ruiliensis*, the forewing has a white dot at middle of termen; the bifurcate part of the uncus is straight, the costa has a bundle of strong spines along distal 1/4, and the cornuti are absent.

#### Description.

Adult (Fig. 10). Wingspan 9.0 mm. Head with vertex shining white, frons shining leaden, occiput dark ochreous brown. Labial palpus with basal segment dark ochreous brown on outer surface, light yellow on inner surface; second segment dark ochreous brown on outer surface, basal 2/5 light yellow, distal 3/5 dark yellow on inner surface; third segment black except white at base and apex, slightly shorter than second. Antenna with scape white, pecten dark brown; flagellum white and black on dorsal surface, dark brown on ventral surface. Thorax and tegula dark ochreous brown. Forewing ochreous brown, sporadically with black scales; markings white sparsely edged with black scales; costal margin black along basal 1/4, with a large spot at 2/3 crossing 3/5 width; three white streaks arising from dorsum: basal streak relatively thin, from dorsal 1/5 to near costal margin, interrupted anteriorly, second streak from dorsal 1/3 to basal 1/3 of upper margin of cell, third streak from dorsal 3/4 extending to before lower angle of cell; apex white; cilia greyish brown, white on apex. Hindwing and cilia greyish brown.

**Male genitalia** (Fig. 26). Uncus with basal 1/2 nearly quadrate; distal 1/2 bifurcate, forming two horn-shaped lateral processes, curved ventrad, sinuate, tapering to pointed apex. Gnathos subtriangular, membranous, sclerotized laterally. Tegumen broad, branched from posterior 1/3, narrowed anteriorly. Valva subtriangular; costa concave at base, projected at 1/5; apex pointed, directing dorsad; ventral margin densely setose on distal 1/2. Sacculus broad at base, gradually narrowed to distal end. Saccus triangular, about 1/2 length of uncus. Juxta broad, with a small saccate basal process; lateral lobes short and broad, somewhat semicircular, reaching near middle of tegumen. Aedeagus gently curved, about 1.5 times length of valva; numerous short spinose cornuti present along 3/5 distal part of the aedeagus.

**Female.** Unknown.

#### Distribution.

China (Xizang).

#### Etymology.

The specific name is derived from Latin *spinosus* (= spinose), and *cornutus*, referring to the numerous cornuti.

### Promalactis strumifera sp. n.

urn:lsid:zoobank.org:act:E07B58C0-2BEA-423A-B7F8-09608A6305C7

http://species-id.net/wiki/Promalactis_strumifera

Figs 11 27 35

#### Type material.

Holotype ♂ − **China,** **Zhejiang Province:** Mt. Jiulong (28°21'N, 118°52'E), 400 m, 5.VIII.2011, coll. Linlin Yang & Na Chen, genitalia slide No. DZH12050 (NKU). Paratypes − 1 ♂, same data as holotype except dated 4.VIII.2011; **Zhejiang Province,** Wuyanling (27°42'N, 119°39'E), Taishun County: 1 ♂, 400 m, 1.VIII.2005, coll. Yunli Xiao; 4 ♂, 680 m, 28.VII−2.VIII.2005, coll. Yunli Xiao; 2 ♂, 790 m, 2, 3.VIII.2007, coll. Qing Jin. **Guangdong Province:** 2 ♂, 1 ♀, Nanling (23°20'N, 115°23'E), Shaoguan City, 7−14.VII.2007, coll. Min Wang *et al*. **Fujian Province:** 9 ♂, 4 ♀, Sangang (27°45'N, 117°40'E), Mt. Wuyi, 740 m, 26, 27.VII.2008, coll. Weichun Li, Yongling Sun & Haiyan Bai. **Guangxi Zhuang Autonomous Region:** 3 ♀, Qinmu village, Yongfu County (24°59'N, 109°59'E), 160 m, 5.V.2008, coll. Li Zhang & Hui Zhen; 1 ♀, Hongqi Forest Farm, Shangsi County (22°09'N, 107°58'E), 260 m, 2.IV.2002, coll. Shulian Hao & Huaijun Xue; 1 ♀, Fubo Forest Farm, Pingxiang City (22°07'N, 106°44'E), 550 m, 1.VIII.2011, coll. Bingbing Hu *et al*., genitalia slide Nos. W05010 ♂, W05028 ♂, DZH11063 ♀, DZH12051 ♀, DZH12052 ♂ (NKU). **Jiangxi Province:** 1 ♂, Dayu County (25°23'N, 114°22'E), 15.VI.1976; 1 ♀, Dayu County, 14.VIII.1976; 1 ♂, Xingguo County, 19.VII.1976. **Hunan Province:** 1 ♂, Suoxiyu (29°35'N, 110°57'E), 17.X.1988, genitalia slide Nos. DZH12033 ♂, DZH12034 ♀(IOZ).

#### Diagnosis.

This species is similar to *Promalactis fascispinata* Du, Li & Wang, 2011, but can be separated by the rectangular gnathos, the dorsal lobe of the valva bifurcate distally and the ventral lobe with two digitate distal processes, and the juxta without spines in the male genitalia. In *Promalactis fascispinata*, the gnathos is tongue shaped, the dorsal lobe of the valva is not bifurcate and the ventral lobe has two elongate ovate distal processes, and the juxta has an ovate cluster of fine spines distally in the male genitalia.

#### Description.

Adult (Fig. 11). Wingspan 8.0−11.5mm. Head with vertex shining white, frons shining leaden, occiput dark ochreous brown. Labial palpus with basal and second segments yellow on inner surface, ochreous brown on outer surface; third segment black, almost same length as second. Antenna with scape white; flagellum white except several distal flagellomeres dark brown on dorsal surface, dark brown on ventral surface. Thorax and tegula dark ochreous brown. Forewing ground colour ochreous brown tinged with dark ochreous brown, sometimes scattered with black scales on lower angle of cell; costal margin greyish black along basal 3/4, with a large rounded white spot at distal 1/4, slightly across middle of wing, edged with dense black scales except on anterior margin; two parallel oblique white streaks arising from dorsum, edged with dense black scales: basal streak from dorsal 1/5 to base of fold, second streak from beyond middle of dorsum to basal 1/3 of upper margin of cell, area ferrugineous between two streaks; dense black scales extending from apex along termen to tornus, forming a narrow black apical band; cilia yellow, dark greyish brown along distal part of costal margin, dark grey along distal part of dorsal margin. Hindwing and cilia grey.

**Male genitalia** (Fig. 27). Uncus stout, heavily sclerotized, sinuate marginally, with a heavily sclerotized, short, triangular apical process at middle; basal 2/3 open ventrally. Gnathos heavily sclerotized, rectangular, densely with warts, blunt at apex: right side concave in U shape near apex; lateral arm almost same length as gnathos, broad, nearly semicircular basally. Tegumen narrowly elongate, almost parallel laterally, branched from posterior 3/10, blunt anteriorly. Valva narrow, almost parallel dorso-ventrally; costa projected at middle, concave near apex; apex bilobed: dorsal lobe short and sclerotized, bifurcate distally, forming two thick spines, dorsal spine short, about 1/3 length of ventral spine, with a brush of setae between two spines; ventral lobe elongate, about 1.4 times length of dorsal lobe, weakly sclerotized, very narrow basally, broadened gradually, distally setose, bifurcate, forming two slender, digitate processes: dorsal process straight, ventral process slightly shorter than dorsal process, curved dorsad distally. Sacculus with basal 3/5 broad and almost parallel sided, distal 2/5 gradually narrowed to base of ventro-apical lobe of valva. Saccus slightly shorter than uncus, somewhat semi-oval. Juxta strong, rod-like, curved dorsad at basal 1/3, with a small awl-shaped process at base, apex narrowly rounded or bluntly pointed, reaching near posterior margin of tegumen; diaphragm with large sclerotized rumples dorsally, enlarged and protruded leftward. Aedeagus almost straight, basal 2/9 slender, slightly curved at 2/9; distal 7/9 broad, uniformly thick, apex pointed; cornutus absent.

**Female genitalia** (Fig. 35). Apophysis anterioris stronger, about 1/3 length of apophysis posterioris. Eighth tergum sclerotized, nearly trapezoidal, convex antero-laterally, sinuate and with sparse long setae on posterior margin. Seventh abdominal segment sclerotized, laterally with a nodular process at anterior 2/5, posterior margin serrate, sometimes with large lateral tooth. Ostium bursae heavily sclerotized and large. Lamella postvaginalis with dorsal part broad leaf-like, posterior margin serrate and with sparse setae, produced to a sclerotized, ovate process at middle, margined with small teeth; ventral part with two lateral processes: left process with basal 1/3 narrow, distal 2/3 abruptly broadened, with ten spines of varied length; right process nearly spine-like, slightly curved at base. Lamella antevaginalis heavily sclerotized, very short, nearly band shaped, anterior and posterior margin convex at middle. Antrum very short, nearly funnel shaped. Ductus bursae curved, slightly longer than corpus bursae, membranous, posterior 3/5 thin, with discontinuous, weakly sclerotized bands, anterior 2/5 enlarged, with a weakly sclerotized, thin ring at anterior 2/5; ductus seminalis arising from anterior 2/5 of ductus bursae. Corpus bursae nearly oval, membranous, with dense granules; a small and rounded signum bearing one larger and one smaller conical spines, with a shield-like, weakly sclerotized plate at base.

#### Distribution.

China (Fujian, Guangdong, Guangxi, Jiangxi, Zhejiang).

#### Etymology

**.** This specific name is derived from Latin *strumifer* (= nodular), referring to the lateral nodular process at anterior 2/5 of the 7th abdominal segment in the female genitalia.

### Promalactis uncinispinea sp. n.

urn:lsid:zoobank.org:act:265C422D-84CB-4DC5-9193-C6567821B93E

http://species-id.net/wiki/Promalactis_uncinispinea

Figs 12 28

#### Type material.

**China: Sichuan Province:** Holotype ♂, Mt. Qingcheng (30°58'N, 103°31'E), 16.vii.1980, genitalia slide No. DZH12185 (IOZ).

#### Diagnosis.

This species is extremely similar to *Promalactis serpenticapitata* sp. n. It can be separated by the gnathos with a triangular subapical process ventrally, the distal process of the sacculus with a small dentate dorso-medial process, the juxta with a bundle of setae and short spines in distal 1/3, and the cornutus about 2/3 length of aedeagus in the male genitalia. In *Promalactis serpenticapitata* sp. n., the gnathos has a snake head-shaped subapical process ventrally, the distal process of the sacculus lacks the dorso-medial process, the juxta has a bundle of setae and short spines in distal 7/10, and the cornutus is about 1/3 the length of the aedeagus. This species is also superficially similar to *Promalactis dierli* Lvovsky, 2000, but can be easily separated by the male genitalia with a symmetrical valva and the aedeagus with one cornutus. In *Promalactis dierli*, the valva is asymmetrical and the aedeagus has no cornutus in the male genitalia.

#### Description.

Adult (Fig. 12). Wingspan 11.0 mm. Head with vertex and frons silvery white mixed with brown, occiput dark brown. Labial palpus with basal and second segments dark brown; third segment black except white at base and apex, slightly shorter than second. Antenna with scape white except black on anterior and posterior margins; flagellum white and black on dorsal surface, dark brown on ventral surface. Thorax and tegula dark ochreous brown. Forewing with basal 3/5 ochreous brown, distal 2/5 ochreous yellow; markings silvery white or white, edged with dense black scales; costal margin with a semicircular silvery white spot at middle; cell with a small silvery white dot under costal spot; three silvery white streaks arising from dorsal margin: basal streak to base of fold, second streak from dorsal 1/3 straight to basal 1/3 of upper margin of cell, third streak from dorsal 2/5 obliquely to distal 1/4 of cell on lower margin; fold with a white dot at end; apex with an elliptic white spot, edged with dense black scales; cilia yellow, grey along distal part of dorsal margin. Hindwing and cilia dark grey.

**Male genitalia** (Fig. 28). Uncus subtriangular, broad at base, narrowed to pointed apex, with a subapical tooth ventrally. Gnathos almost same length as uncus, slender, distal 1/4 scobinate and curved ventrad, apex narrowly rounded, ventrally with a small, triangular subapical process; lateral arm band shaped, about 1/4 length of gnathos. Tegumen narrow posteriorly, convex laterally at posterior 1/3, branched from posterior 2/3, rounded apically. Valva sclerotized; basal 2/3 almost parallel dorso-ventrally, distally produced to a setose triangular process; costa with basal 3/5 straight, distal 2/5 concave. Sacculus broad at base, slightly narrowed distally, dorsal margin concave between basal 2/5−2/3, distal 1/3 produced to a free, setose distal process, which bears a small, heavily sclerotized, dentate process dorso-medially, apex pointed and directing dorsad, far exceeding tip of costa. Vinculum widened anteriorly, with a slender transverse band joining left and right sides, forming a fan-shaped area between this band and posterior margin of saccus. Saccus short and broad, slightly shorter than uncus, subtriangular, narrowly rounded at apex. Juxta long, nearly rod-like, slightly curved, broad basally, with a short digitate basal process, distal 1/3 with a bundle of setae and short spines on dorsal surface, apically with dense short spines, reaching near posterior margin of tegumen. Aedeagus straight and short, about 3/5 length of valva; with two pieces of dense microtrichia and a heavily sclerotized hooked spine distally; cornutus slightly curved, basal half weakly sclerotized and rod-like, distal half heavily sclerotized and spine-like, situated at middle, about 2/3 length of aedeagus, with several short spines medially.

**Female.** Unknown.

#### Distribution.

China (Sichuan).

#### Etymology.

The specific name is derived from the Latin prefix *uncin-* (= hooked), and Latin *spineus* (= spine-like), referring to the hooked distal spine in the aedeagus.

**Figures 13–16.**
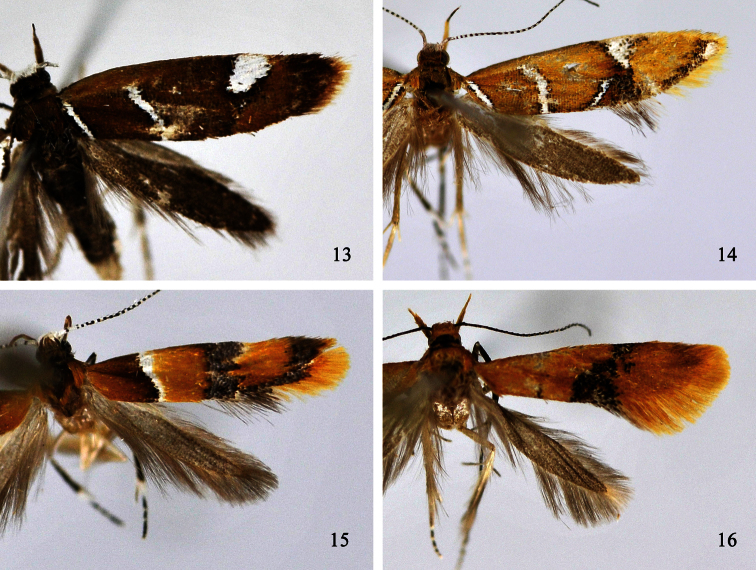
Adults of *Promalactis* species. **13** *Promalactis albipunctata* Park & Park, female **14** *Promalactis dierli* Lvovsky, female **15** *Promalactis dimolybda* Meyrick, male **16** *Promalactis flavescens* Wang, Zheng & Li, female.

**Figures 17–22.**
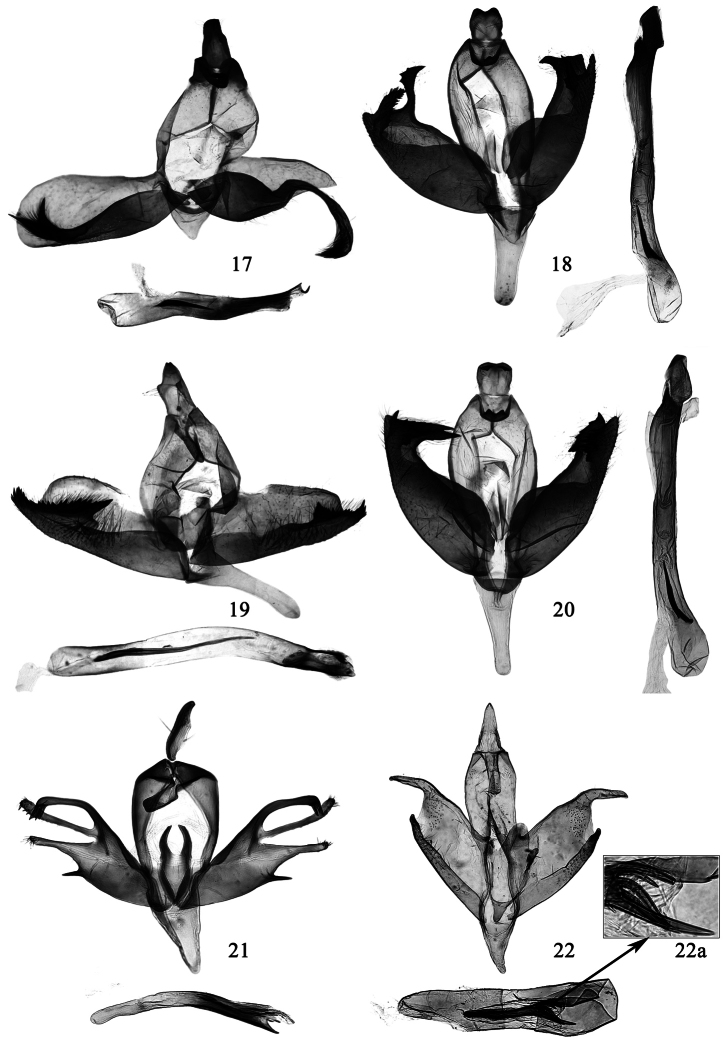
Male genitalia of *Promalactis* species. **17** *Promalactis bifurciprocessa* sp. n., holotype, slide No. DZH12198 **18** *Promalactis convexa* sp. n., holotype, slide No. DZH12027 **19** *Promalactis papillata* sp. n., holotype, slide No. DZH12147 **20** *Promalactis quadratitabularis* sp. n., holotype, slide No. DZH12037 **21** *Promalactis quadriloba* sp. n., holotype, slide No. DZH12032 **22** *Promalactis ramispinea* sp. n., holotype, slide No. DZH11025 **22a** enlarged distal part of cornutus.

### Promalactis albipunctata

Park & Park, 1998

http://species-id.net/wiki/Promalactis_albipunctata

Figs 13 29 36

Promalactis albipunctata Park & Park, 1998: 58. Type locality: Korea (South).

#### Material examined.

**China, Jiangxi Province:** 1♂, 1 ♀, Dayu County, 18.VI.1976; 1 ♀, Mt. Jiulian, 23.V.1977, genitalia slide Nos. DZH12176 ♂, DZH12203 ♀ (IOZ). **Fujian Province:** 1♂, Sangang, Mt. Wuyi, 740 m, 27.VII.2008, coll. Weichun Li, Yongling Sun & Haiyan Bai. **Zhejiang Province:** 1 ♀, Mt. Jiulong, 400 m, 5.VIII.2011, coll. Linlin Yang & Na Chen, genitalia slide Nos. DZH12053 ♂, DZH12054 ♀ (NKU).

#### Diagnosis.

Adult with wingspan 11.0−14.0 mm. This species is similar to *Promalactis parasuzukiella* Wang, 2006, but can be separated by the sacculus with a digitate apical process, the slender rod-like saccus and the aedeagus with two spine-like apical processes in the male genitalia (Fig. 29); the M-shaped lamella postvaginalis and the oval signum in the female genitalia (Fig. 36). In *Promalactis parasuzukiella*, the sacculus has no digitate apical process, the saccus is semi-oval and the aedeagus has no apical process; and the lamella postvaginalis is absent and the signum is cross shaped.

#### Remarks.

*Promalactis albipunctata* was described by Park and Park (1998) on the basis of six female specimens from Korea. *Promalactis akaganea* Fujisawa, 2002 was described from three male and seventeen female specimens from Japan. By checking the photographed adult and both male and female genitalia of *Promalactis albipunctata*, we suspect that *Promalactis akaganea* is a synonym of *Promalactis albipunctata*, which can be confirmed only after checking the types of *Promalactis akaganea*.

#### Distribution.

China (Jiangxi); Korea.

#### Note.

This species is recorded from China for the first time.

### Promalactis dierli

Lvovsky, 2000

http://species-id.net/wiki/Promalactis_dierli

Figs 14 37

Promalactis dierli Lvovsky, 2000: 667. Type locality: Nepal.

#### Material examined.

**China, Xizang autonomous Region:** 1 ♀, Zhangmu Port, 11.VIII.1981, coll. Shengyuan Hu, genitalia slide No. DZH12003 (IOZ).

#### Diagnosis.

Adult with wingspan 14.5 mm. This species is very similar to *Promalactis jezonica* (Matsumura, 1931), but can be separated by the forewing without the white dot at end of the fold (Fig. 14); the uncus having a triangular process at basal 3/5 ventrally, the broad leaf-like juxta, and the aedeagus with a short spine extending from dorsal side at distal 1/4 in the male genitalia (Lvovsky 2000, fig. 5), and further the mound-like lamella postvaginalis in the female genitalia (Fig. 37). In *Promalactis jezonica*, the forewing has a white dot at end of the fold, the uncus lacks the triangular process, the juxta is very slender, and the aedeagus has no distal spine; and the lamella postvaginalis is nearly crown shaped.

#### Distribution.

China (Xizang); Nepal.

#### Note.

This species is recorded from China for the first time.

### Promalactis dimolybda

Meyrick, 1935

http://species-id.net/wiki/Promalactis_dimolybda

Figs 15 30 38

Promalactis dimolybda Meyrick, 1935: 78. Type locality: China (Tien-Mu-Shan).

#### Material examined.

**China,** **Zhejiang Province:** 2 ♂, 33 ♀, Mt. Fengyang, Lishui City, 1470 m, 25−30.VII.2007, coll. Qing Jin. **Fujian Province:** 19 ♀, Guadun, Mt. Wuyi, 1100 m, 28.VII−2.VIII.2008, coll. Weichun Li, Yongling Sun & Haiyan Bai. **Hubei Province:** 1 ♀, Houhe, Wufeng County, 1100 m, 11.VII.1999, coll. Houhun Li *et al*., genitalia slide Nos. W00106 ♀, ZL08133 ♂, DZH08042 ♀, DZH08043 ♀, DZH08044 ♂, DZH08046 ♀, DZH12039 ♀, DZH12040 ♀, DZH12112 ♀ (NKU); **Sichuan Province:** 3 ♂, 3 ♀, Wanniansi, Mt. Emei, 14.VI.1979, genitalia slide Nos. DZH12007 ♀, DZH12008 ♂, DZH12041 ♂ (IOZ).

#### Diagnosis.

This species is similar to *Promalactis taibaiensis* Wang, Zheng & Li, 1997, but can be separated by the aedeagus with two apical spines and two cornuti in the male genitalia; the ductus bursae concave ventrally at middle on posterior margin and membranous between posterior 3/5−3/4, and the signum with small distinct or indistinct teeth on posterior end in the female genitalia. In *Promalactis taibaiensis*, the aedeagus has four apical spines and one cornutus; the ductus bursae is slightly convex ventrally on posterior margin and entirely sclerotized, and the signum has dense teeth.

#### Redescription.

Adult (Fig. 15). Wingspan 9.5−11.5 mm. Head with vertex shining white, frons shining leaden grey, occiput yellowish brown. Labial palpus with basal and second segments yellow, third segment dark brown, almost same length as second. Antenna with scape white; flagellum white and black on dorsal surface, dark brown on ventral surface. Thorax and tegula ochreous brown. Forewing ground colour ochreous yellow; a narrow white fascia from costal 1/4 to dorsal 2/5, its inner margin edged with dense black scales, area ochreous brown from inner margin to base; a broad dark grey fascia at 3/5, tinged with black scales, its inner margin straight, outer margin sinuate; a wedge-shaped dark grey fascia from apex of costal margin along termen to end of fold, tinged with black scales; a narrow dark grey band along dorsal margin between two dark fasciae and connected them; cilia yellow, dark grey along distal part of costal margin, grey along distal part of dorsal margin. Hindwing and cilia dark grey.

**Male genitalia** (Fig. 30). Uncus nearly bell shaped, broad at base, gradually narrowed to 3/5, distal 2/5 slender, rounded at apex, laterally with setae. Gnathos tongue shaped, about 2/3 length of uncus, distal 1/2 scobinate, apex broadly rounded; lateral arm short, band shaped, about 1/3 length of gnathos. Tegumen branched from posterior 1/2, very narrow anteriorly. Valva narrowed and setose distally, apex narrowly rounded and directing dorsad; costa sinuate, concave at base and before apex, projected at middle. Sacculus narrow, slightly concave at basal 3/5 on dorsal margin, distal 2/5 setose; distal 1/5 free, serrate dorsally; apex pointed, directing dorsad, not reaching end of valva. Saccus slender, rod-like, slightly broader at base, rounded at apex, almost as long as valva. Juxta weakly sclerotized, short, with a small, slender awl-shaped basal process; lateral lobes broad, irregularly quadrate, rounded at apex, reaching middle of tegumen. Aedeagus gently curved, dilated distally, with two curved, basally joined distal spines; two joined or separate, spine-like cornuti present at middle: one very small, the other larger, sometimes deciduate.

**Female genitalia** (Fig. 38). Apophysis anterioris stronger than and about 1/2 length of apophysis posterioris. Ductus bursae about twice length of corpus bursae, posterior margin ventrally concave at middle and protruded laterally, posterior 3/5 sclerotized and sinuate, with some spinules at posterior 3/5, posterior 3/5−3/4 membranous and expanded, anterior 1/4 sclerotized, curved in semi-volute or sinuate; ductus seminalis arising from posterior 2/3 of ductus bursae. Corpus bursae rounded; signum small, nearly oval or rhombic, with small distinct or indistinct teeth on posterior end.

#### Distribution.

China (Fujian, Hubei, Sichuan, Zhejiang).

**Note.** Themale is described for the first time.

### Promalactis flavescens

Wang, Zheng & Li, 1997

http://species-id.net/wiki/Promalactis_flavescens

Figs 16 39

Promalactis flavescens Wang, Zheng & Li, 1997: 202; Wang 2006: 32. Type locality: China (Shaanxi).

#### Material examined.

**China,** **Sichuan Province:** 4 ♂, 2 ♀, Mt. Qingcheng, 19−24.V.1979; 1 ♂, 1 ♀, Wanniansi, Mt. Emei, 14.VI.1979, genitalia slide Nos. DZH12042 ♂, DZH12014 ♀ (IOZ).

#### Diagnosis.

Adult with wingspan 12.5−14.0 mm. This species is similar to *Promalactis bitaenia* Park & Park, 1998, but can be separated by the forewing with a dark brown fascia (Fig. 16); the sacculus with a bundle of strong setae on the dorsal margin distally, the aedeagus about twice length of the valva and with a small subapical tooth in the male genitalia (Wang 2006, fig. 40); the lamella postvaginalis with the dorsal part nearly quadrangular and the ventral part trapezoidal in the female genitalia (Fig. 39). In *Promalactis bitaenia*, the forewing has two dark brown fasciae; the sacculus has spines and small teeth on dorsal margin distally, and the aedeagus is slightly longer than the valva and lacks the subapical tooth; and the lamella postvaginalis is irregularly rounded.

**Female genitalia** (Fig. 39). Apophysis anterioris about 1/2 length of apophysis posterioris. Eighth tergum with sparse long setae on posterior margin. Lamella postvaginalis heavily sclerotized, dorsal part elongate, nearly quadrangular, rounded on posterior margin, ventral part short, about 3/5 length of dorsal part, trapezoidal; lamella antevaginalis large, anterior 3/5 broad and slightly convex laterally, narrowed near anterior margin, anterior margin straight, triangularly protruded backward at anterior 3/5 laterally. Antrum elongate, tubular, posterior 1/3 broader. Ductus bursae weakly sclerotized except basal small portion heavily sclerotized, membranous near corpus bursae, with two plates near middle: one plate hand shaped, protruded, with three strong, curved spines on anterior edge; the other plate subtriangular, with very short spines on anterior edge. Corpus bursae small, rounded and membranous; signum absent.

#### Distribution.

China (Shaanxi, Sichuan).

#### Note.

The female of this species is described here for the first time.

**Figures 23–28.**
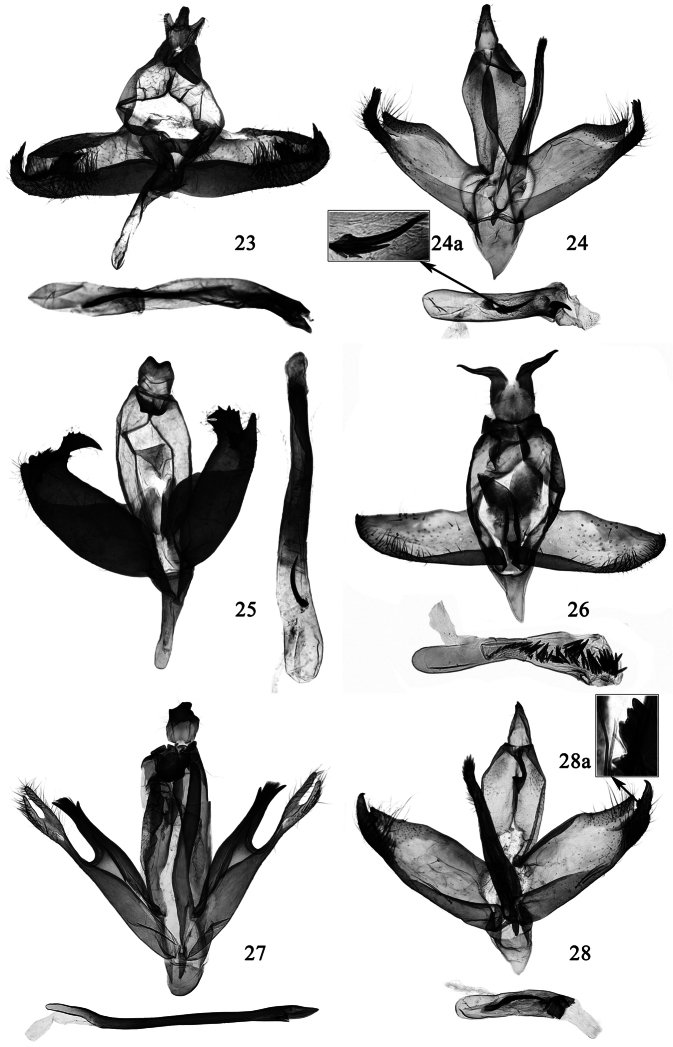
Male genitalia of *Promalactis* species. **23** *Promalactis scorpioidea* sp. n., paratype, slide No. DZH12012 **24** *Promalactis serpenticapitata* sp. n., paratype, slide No. DZH12046 **24a** enlarged cornutus **25** *Promalactis similiconvexa* sp. n., holotype, slide No. DZH12178 **26** *Promalactis spinosicornuta* sp. n., slide No. DZH12011 **27** *Promalactis strumifera* sp. n., paratype, slide No. DZH12052 **28** *Promalactis uncinispinea* sp. n., holotype, slide No. DZH12185 **28a** enlarged dentate dorsal process of sacculus.

**Figures 29–33.**
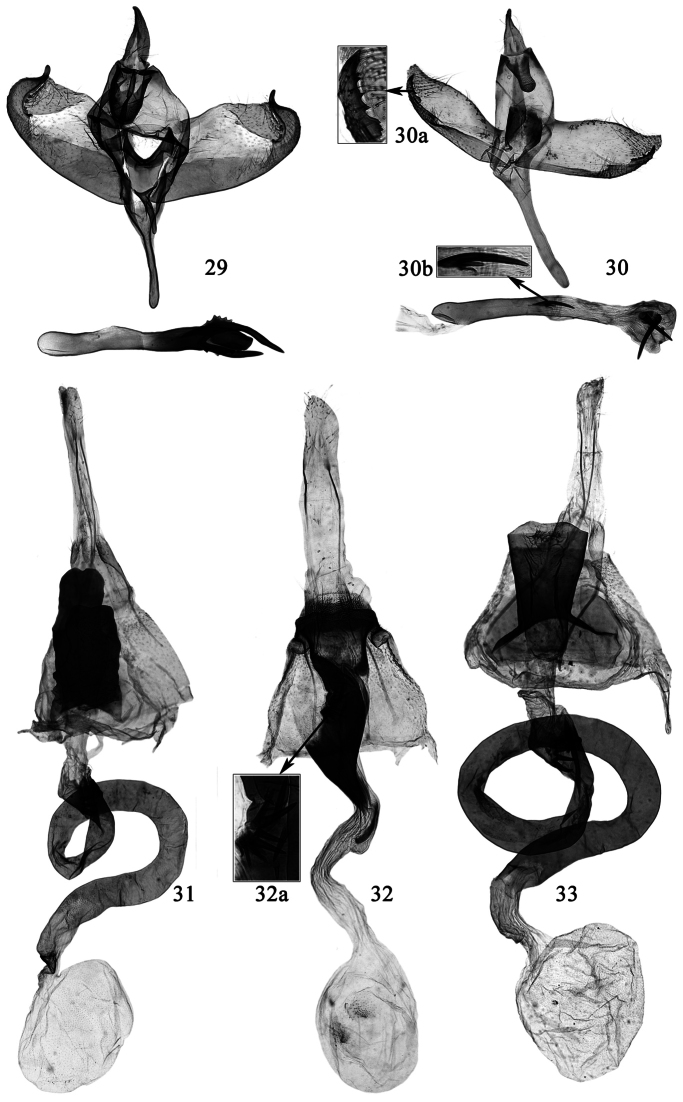
**29–30** Male genitalia of *Promalactis* species. **31–33** Female genitalia of *Promalactis* species **29** *Promalactis albipunctata* Park & Park, slide No. DZH12176 **30** *Promalactis dimolybda* Meyrick, slide No. DZH12041 **31** *Promalactis papillata* sp. n., slide No. DZH12196 **32** *Promalactis ramispinea* sp. n., slide No. DZH12013 **32a** enlarged spines of ductus bursae **33** *Promalactis scorpioidea* sp. n., slide No. DZH12189.

**Figures 34–39.**
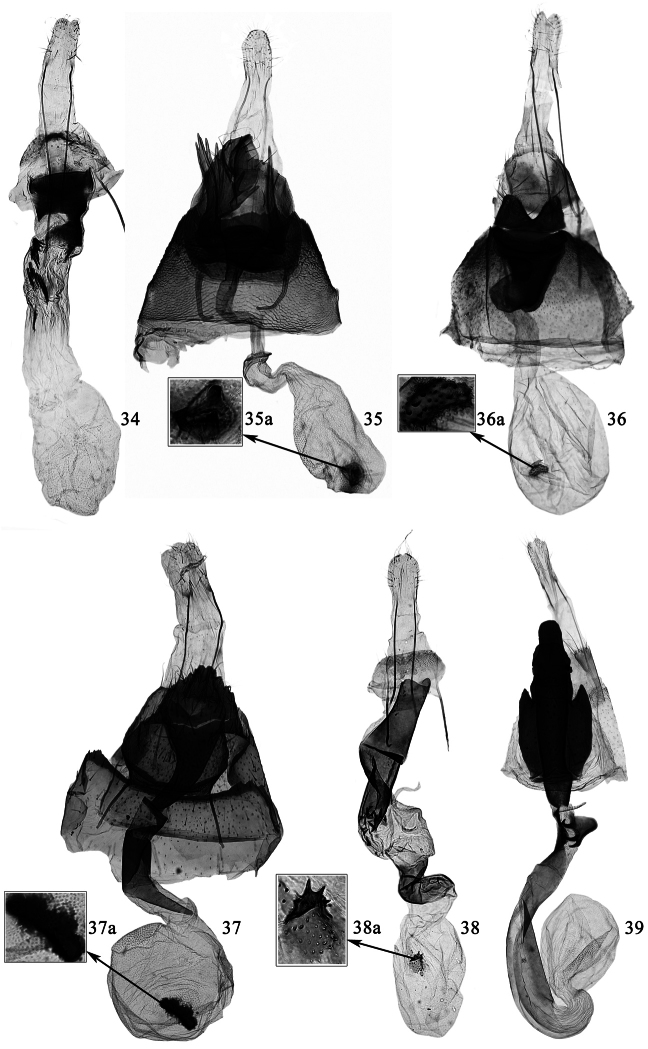
Female genitalia of *Promalactis* species. **34** *Promalactis serpenticapitata* sp. n., slide No. DZH12038 **35** *Promalactis strumifera* sp. n., slide No. DZH12034 **35a** enlarged signum **36** *Promalactis albipunctata* Park & Park, slide No. DZH12203 **36a** enlarged signum **37** *Promalactis dierli* Lvovsky, slide No. DZH12003 **37a** enlarged signum **38** *Promalactis dimolybda* Meyrick, slide No. DZH12039 **38a** enlarged signum **39** *Promalactis flavescens* Wang, Zheng & Li, slide No. DZH12014.

## Supplementary Material

XML Treatment for Promalactis bifurciprocessa

XML Treatment for Promalactis convexa

XML Treatment for Promalactis papillata

XML Treatment for Promalactis quadratitabularis

XML Treatment for Promalactis quadriloba

XML Treatment for Promalactis ramispinea

XML Treatment for Promalactis scorpioidea

XML Treatment for Promalactis serpenticapitata

XML Treatment for Promalactis similiconvexa

XML Treatment for Promalactis spinosicornuta

XML Treatment for Promalactis strumifera

XML Treatment for Promalactis uncinispinea

XML Treatment for Promalactis albipunctata

XML Treatment for Promalactis dierli

XML Treatment for Promalactis dimolybda

XML Treatment for Promalactis flavescens
